# Notes on Preparations for the Sick

**Published:** 1897-12

**Authors:** 


### Notes on Preparations for the Sick.

**Lactophenin.**—C. F. Boehringer & Soehne, Mannheim.—We have received from Messrs. Parke, Davis & Co. a sample of this preparation. The drug is a phenacetin, in which the acetic acid is replaced by lactic acid. It is more soluble, and is said to be preferable on account of its relative freedom from toxic action. The ordinary dose is ten grains, but as much as thirty grains per dose or seventy-five grains per day may be safely given. Phenacetin is a drug which usually acts efficiently, and rarely with any disagreeable toxic results; it has served us so well that there has been little need to look for anything better, and it requires much evidence to convince us that anything better has been found.

**Anti-syphilitic Serum.**—Burroughs, Wellcome & Co., London.—This serum has been prepared from animal blood previously modified by syphilitic injection. The serum is furnished both in the liquid form and as a dry powder. It has been favourably reported on by Dr. A. S. Barling.

**Vitalia Meat Juice.**—The Vitalia Co., London.—This new variety of meat juice is a raw meat preparation in which the character of rawness is not especially obvious in either colour, taste, or odour. The promoters claim for it that it is a distinct advance on any preceding similar preparation, and that it contains a very much larger proportion of proteid material (20 °/₀) than is usually met with in meat juice, whilst it is perfectly sterilised and contains no objectionable preservative. We find it to be a very viscid fluid, of high gravity: after diluting to one in ten the gravity was found to be 1012. The amount of soluble albumen is very high: as estimated by the picric tube after dilution it gave something like twelve per cent.

The raw meat taste is in some way modified so as not to be easily identified: there is nothing unpleasant in the appearance or taste of the fluid, and as it has about 10 °/₀ of added salt and is well sterilised, it needs no alcohol or glycerine for its preservation.

We have not met with a more concentrated liquid meat food than this, and we see no reason why it should not be enjoyed by the invalid who requires the maximum of nourishment in the minimum of bulk.

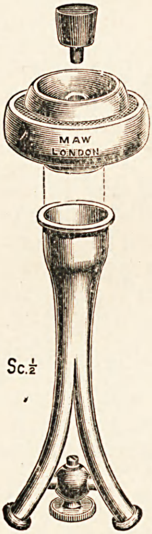

**The Stethonoscope.**—S. Maw, Son, & Thompson, London.—This apparatus is intended for use with the ordinary binaural stethoscope, to which it is easily attached by the India-rubber tubes. It is constructed with a vibrating plate insulated by means of rubber, somewhat on the principle of the microphone. By its use both heart and lung sounds are much more pronounced than when the ordinary stethoscope is used without it. It is preferable to the phonendoscope in that it can be easily carried in the waistcoat pocket and attached to the stethoscope at will. A little tap between the bifurcation allows air to enter or to be excluded from the tubes; but we have not found any particular advantage from this tap, although the instrument as a whole is a great improvement on the ordinary stethoscope, and it must be an advantage to those who from commencing deafness have a difficulty in hearing the ordinary sounds without it.

